# Developing Vaccines to Combat Pandemic Influenza

**DOI:** 10.3390/v2020532

**Published:** 2010-02-02

**Authors:** James S. Robertson, Othmar G. Engelhardt

**Affiliations:** Division of Virology, National Institute for Biological Standards and Control, Health Protection Agency, Blanche Lane, South Mimms, Potters Bar, EN6 3QG, UK; E-Mail: othmar.engelhardt@nibsc.hpa.org.uk (O.G.E.)

**Keywords:** pandemic influenza vaccines, reverse genetics, H5N1 virus, H1N1v virus

## Abstract

Influenza vaccine manufacturers require antigenically relevant vaccine viruses that have good manufacturing properties and are safe to use. In developing pandemic vaccine viruses, reverse genetics has been employed as a rational approach that can also be used effectively to attenuate the highly virulent H5N1 virus and at the same time place the H5 HA and N1 NA on a background of PR8, a virus that has been used over many decades to provide high yielding vaccine viruses. Reverse genetics has also been used successfully alongside classical reassorting techniques in the development of (swine flu) pandemic A(H1N1)v vaccine viruses.

## 1. Introduction

Influenza vaccines were first developed over 60 years ago [1]. They were based upon growth of virus in embryonated hens’ eggs and inactivation of the virus. Little has changed over the decades in this respect so that even now the vast bulk of vaccine being used to combat H1N1v pandemic influenza is the same inactivated egg-derived vaccine. What has taken place is a fine tuning of the manufacturing process with the principal changes concerning the extent of purification and treatment of the virus antigen in order to have a less reactogenic vaccine. Nowadays most inactivated influenza vaccine is a detergent split or a purified subunit vaccine although some whole virion vaccine is still in use [2]. This review does not address the manufacture of inactivated influenza vaccine or the many alternative approaches to an influenza vaccine that are being actively investigated such as live attenuated vaccines, recombinant vaccines, cell-derived inactivated vaccines and many other novel approaches. The scope of this review is to describe the development of the viruses that are required by the vaccine industry in order for them to manufacture an egg-derived inactivated vaccine. These are a crucial component of vaccine production; without a suitable virus, vaccine production would be severely curtailed.

## 2. High Yielding/High Growth Vaccine Viruses

**Figure 1.**
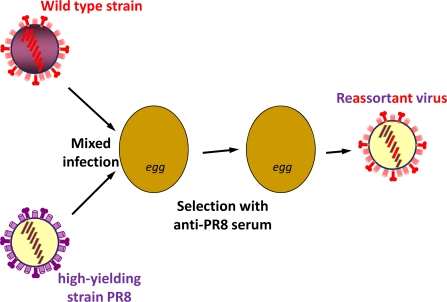
Diagrammatic representation of the classical reassortment of influenza viruses.

Human influenza viruses do not readily grow in embryonated hens’ eggs; they have to adapt to this method of propagation [3–5]. This was first realised during the 1940s and, once adaptation has occurred, it is a highly efficient method of preparing large quantities of influenza virus [1]. The work of E.D. Kilbourne in the 1960s was very important for influenza vaccine production [6,7]. He demonstrated that by infecting an egg with two separate viruses, one a high yielding strain in eggs such as the well used PR8 virus and the other a wild type virus of low egg passage, a progeny virus could be selected from the mixed population of recombinants with the high yielding phenotype of PR8 and the surface antigens of the wild type virus (Figure 1). Selection for viruses with the wild type surface antigens was achieved using anti-PR8 antiserum and selection for high growth was achieved by passage at limit dilution. The intent was that such recombinant viruses would be useful for the efficient manufacture of influenza vaccine. This indeed was the case and vaccine derived from a genetically recombined virus was shown to have the appropriate immunogenicity in humans [8].

This process has been used to this day in deriving vaccine viruses and is undertaken by a small network of laboratories working under the umbrella of the World Health Organisation (WHO) whenever a new influenza A strain is recommended for inclusion in vaccine. Whilst it was suspected early on that the genome of influenza viruses was not a single nucleic acid molecule, it was some time after the demonstration that influenza viruses will readily recombine that it was definitively shown that the genome consists of eight specific segments of RNA; thus this high level of recombination was in fact due to ‘reassorting’ of the genome segments during a co-infection [6,9,10]. The high yielding recombinants (now known as reassortants) first described by Kilbourne typically (but not always) have six segments derived from PR8 (generally termed the internal genes) and two segments encoding the surface HA and NA antigens from the wild type virus; these are termed 6:2 reassortants. Studies have shown that at minimum, high growth reassortants (HGR) based upon PR8 had the PR8 segment encoding the matrix protein [11]. Whilst this process of deriving an HGR remains an essential element of influenza vaccine production, it is highly serendipitous and successful derivation of a useful HGR within the short time frame of the annual influenza vaccine manufacturing season is not guaranteed.

## 3. Reverse Genetics

**Figure 2.**
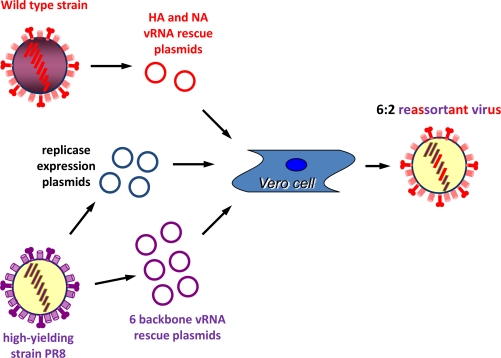
Diagrammatic representation of the reverse genetics process in generating a 6:2 reassortant virus containing the six internal genome segments from PR8 and two genome segments (the HA and NA encoding segments) from a wild type virus.

In more recent years, technology known as reverse genetics has allowed a rational approach to the development of a candidate vaccine virus [12–14]. Reverse genetics involves cloning the individual RNA genome segments of the virus into bacterial plasmids under the control of a mammalian DNA dependent RNA polymerase I (polI)-dependent promoter. Expression of these plasmids in an appropriate mammalian cell line generates exact copies of the viral genome segments. With the cotransfection of the cells with plasmids that express the replicase activity of the virus (PA, PB1, PB2 and NP), infectious virus can be reconstituted (or rescued) (Figure 2).

The way in which this technology can be used to generate potential high growth reassortants is that a virus can be reconstituted specifically with the six internal genome segments of PR8, the high yielding strain, and the two genome segments encoding the HA and NA surface antigens of a wild type virus to create a specific 6:2 reassortant. This is the gene constellation of many classically derived HGRs and studies show that reverse genetics derived 6:2 viruses have growth characteristics comparable to classically derived reassortants [15]. From a practical point of view, the six internal PR8 gene segments can be prepared in advance and used as and when required; similarly for the four plasmids expressing the helper replicase function. Only the two surface antigen segments from the new wild type strain need be cloned at the point of time when a new vaccine strain is required. Other investigators have designed rescue systems based upon eight plasmids and some on even fewer plasmids or other vectors, but essentially they all operate on the same principle of expressing intra-cellular full length copies of the required eight viral genome segments and helper replicase function [16–18]. Thus reverse genetics can be used for the rational design of a reassortant influenza vaccine virus.

There are two additional advantages of this approach. Firstly, clinical samples from patients infected with influenza may contain other viruses with which a patient is co-infected which might replicate in eggs alongside the wild type influenza virus and remain during the derivation of an HGR by the classical approach. Extracting and cloning the RNA segments of an influenza virus during the reverse genetics approach eliminates this risk due to the processes involved and the specificity of PCR used during cloning. Furthermore, for a reverse genetics derived vaccine virus, the HA and NA segments could derive from a wild type virus isolated in cell cultures that have not been validated to be free of endogenous viral contamination; again, the reverse genetics process would eliminate any such infectious material. The second advantage is that when the viral genome is being cloned, the genetic sequences can be modified in a rational manner to introduce advantageous features into the virus that is being rescued. This feature came to the fore when the threat of a pandemic due to H5N1 avian influenza appeared on the horizon in early 2004.

## 4. Use of Reverse Genetics to Derive Safe H5N1 Candidate Vaccine Viruses

Due to the highly pathogenic features of the avian H5N1 virus, it was not practical from a safety point of view to use the wild type virus in an egg production environment. Further, although the virus itself did not have to undergo an egg-adaptation step, being of avian origin in the first place, achieving optimal growth was technically demanding because the virus was highly virulent within the embryonated egg which caused considerable tissue destruction and subsequently it was difficult to purify virus from the fluids. The major molecular basis for the highly pathogenic phenotype resides within the HA genome segment and so reassorting by classical techniques would not necessarily have resulted in a safe virus, even though the intention would have been to introduce as many PR8 backbone segments into a reassortant virus as could be achieved. Thus it was by using reverse genetics that a safe and useful 6:2 reassortant virus could be created.

The molecular feature of high pathogenicity is a short stretch of basic amino acids located within the HA structure at a point known as the cleavage site (the HA has to be cleaved by a proteolyic enzyme in order for the virus to be infectious) [19,20]. The vast majority of influenza virus strains are of low pathogenicity and contain a single arginine residue at the cleavage site. These HAs are cleaved by a trypsin-like protease found generally at epithelial cell surfaces which contributes to the tropism of the virus [21]. In highly pathogenic strains (limited to haemagglutinins of the H5 and H7 subtypes) an extra 3–5 basic (arg or lys) amino acids have been inserted (purportedly by polymerase stuttering during replication of the genome segment). The presence of these extra amino acids allows ubiquitous intracellular subtilisin-like proteases to cleave the HA and results in an altered tropism of the virus such that it can cause a systemic and highly pathogenic infection [21]. During cloning of the HA from a highly pathogenic H5 or H7 virus, it is technically straightforward to remove the region of nucleic acid encoding these amino acid residues and return the HA to a non-pathogenic phenotype [22].

At the onset of the avian H5N1 pandemic threat, there was a call from WHO to the small network of laboratories with this technology to develop appropriate high yielding and safe candidate vaccine viruses. This was achieved rapidly by three laboratories including ours in which the vaccine virus NIBRG-14 was generated within three weeks [15,23]. NIBRG-14 contains the six internal genome segments from PR8 and the HA and NA genome segments from a human H5N1 isolate, A/Vietnam/1194/2004. However, these H5N1 candidate viruses could not be distributed to the vaccine manufacturing industry until it had been demonstrated that they were indeed no longer highly pathogenic. This was achieved by a number of assays [15]. Firstly, sequencing was used to demonstrate that the HA genome segment of NIBRG-14 had the deletion it was designed to have. Secondly, growth of influenza viruses in cell culture typically requires the addition of trypsin to cleave the HA to form infectious particles. In the case of the highly pathogenic influenza viruses, there is no requirement for trypsin because endogenous proteases in the cell cultures will perform this action. Thus the highly pathogenic phenotype can be assayed for by assessing the ability of a virus to form infectious centres in the presence or in the absence of added trypsin. A virus of the non-pathogenic phenotype will form infectious centres only in the presence of trypsin. This was found to be the case for NIBRG-14 confirming the non-pathogenic nature of the virus. Three *in vivo* assays were also utilised. The wild type H5N1 viruses cause embryo death after inoculation of hens’ eggs. The 6:2 reassortant NIBRG-14 did not cause embryonic death indicating that the virus was attenuated compared to the wild type. In international veterinary surveillance of avian viruses, the *in vivo* chicken pathogenicity (IVCP) test is a well established assay stipulated by the Office International des Epizooties to determine the level of pathogenicity of H5 and H7 isolates [24]. The most pathogenic H5 and H7 viruses score a maximum of 3.0 in the IVCP assay whilst a value of <1.2 is indicative of a virus of low pathogenicity. NIBRG-14 scored 0.0 in this assay confirming again the highly attenuated nature of the virus.

Because of the novelty of this situation, however, there remained concern regarding the pathogenicity of such viruses for mammalian species. The ferret is the mammalian model of choice for *in vivo* studies of influenza viruses and a ferret assay was adopted by the WHO as the standard test to be used in assessing the mammalian pathogenicity of candidate vaccine viruses that have been derived from pathogenic wild type viruses [25]. NIBRG-14 was inoculated intranasally into four ferrets alongside the wild type virus and PR8 which is highly attenuated for humans. In this assay, the pathogenicity of NIBRG-14 as determined by virus production in the respiratory tract and in specified organs, and by histological examination, was similar to the highly attenuated PR8 and considerably less than that of its wild type parental H5N1 virus (A/Vietnam/1194/2004) which was highly pathogenic and caused systemic infection. Consequently NIBRG-14 was deemed safe and suitable for pandemic influenza vaccine manufacture. Many influenza vaccine manufacturers have used NIBRG-14 to prepare pandemic-like vaccines for investigative purposes and in Europe some have used such studies with NIBRG-14 to gain regulatory approval of an H5N1 vaccine as part of the ‘core’ dossier regulatory approach developed by the EMEA as a fast track procedure for pandemic vaccine approval [26–30].

Since 2004, H5N1 has remained a major pandemic threat. As is typical for influenza viruses, as the H5N1 panzootic developed the viruses have undergone genetic and antigenic drift and many clades and sub-clades which are antigenically distinct have been identified [31]. The network of WHO laboratories able to use reverse genetics to create candidate vaccine viruses has continued to respond to the emergence of new H5N1 variants and since the start of the panzootic at least 17 candidate vaccine viruses have been developed, all by reverse genetics technology (Table 1)[31]. Vaccine manufacture also requires virus-specific reagents to measure and standardise the antigen content of vaccine [32]. These reagents consist of a vaccine virus-specific antigen of calibrated content and an appropriate anti-antigen antiserum. These reagents are used commonly in the single radial immunodiffusion (SRD) assay for quantification of HA antigen in vaccine or at different stages of the manufacturing process. Such reagents have also been created for many of the candidate H5N1 vaccine viruses and many are available from NIBSC [33].

When the NIBRG-14 virus began to be used in vaccine manufacture, the vaccine industry noted that this and other H5N1 strains gave suboptimal yields of antigen. This caused concern in that the projected supply of vaccine in the event of a pandemic was going to be considerably less than previously estimated. At NIBSC, investigations were undertaken to determine the nature of the reduced antigen yield. Virus growth did not seem to be compromised as the virus could be grown to a reasonable titre in eggs; however, in the process of calibrating the reagents for vaccine potency, it was noticed that the HA content of harvested virions appeared to be less than normal. HA content of a virus preparation is typically determined from a PAGE analysis of the virus run under non-reducing or reducing conditions, followed by densitometric evaluation of the percentage HA of total virion protein. For the H5N1 viruses and vaccine viruses containing the H5N1 surface antigens, there were technical problems with PAGE analysis. Under non-reducing conditions, high molecular weight aggregates of protein were observed which contained HA, making assessment of the principal HA band unreliable, whilst under reducing conditions, the HA1 moiety ran adjacent to the NP protein and the HA2 moiety ran adjacent to the M1 protein, again making densitometric measurements potentially unreliable. This problem was overcome by the use of an endoglycosidase digestion step to remove the carbohydrate moieties from the HA glycoprotein [34]. This had two effects, firstly it caused the HA1 and the HA2 to run as tighter bands in reduced PAGE and secondly it reduced the molecular weight of these proteins so that they migrated faster into a region of the gel clear of other viral proteins and thus densitometric analysis could be applied with greater reliance on the quantitative measurement. Analysis of NIBRG-14 in this way confirmed that the HA content was only 78% that of virus PR8 and would contribute to the low yield of antigen being derived from this and other H5N1 vaccine candidate viruses [34].

Studies are underway at NIBSC to improve the HA content of NIBRG-14 virions and will be the subject of a separate publication. Other laboratories have reported on approaches to improve pandemic candidate vaccine viruses, for example, by use of alternate PR8 backbone strains, by creating 7:1 reassortants in which only the HA is derived from the wild type virus, or by genetic modification of the neuraminidase [35,36].

## 5. Library of Viruses of Pandemic Potential

Highly pathogenic avian H5N1 viruses are not the only pandemic threat, as other animal viruses have occasionally infected humans and other mammals causing disease ranging from mild symptoms to fatal outcome [37–39]. While none of these viruses has yet been shown to transmit efficiently from human to human, it is difficult to determine the degree of pandemic risk posed by animal influenza viruses of various subtypes. Therefore, pandemic preparedness should be extended to viruses beyond the highly pathogenic H5N1 viruses. This could include the generation of candidate vaccine viruses from viruses that are considered to be of pandemic concern before they emerge as novel human pathogens, *i.e.* in the pre-pandemic period. Consequently, and due to the large number of potential viruses that could be subsumed under this category, a ‘library’ of candidate vaccine viruses can be envisaged as one component in the global pandemic preparedness tool kit. This library is made up of viruses representing various subtypes and will hopefully cover most animal influenza viruses that may cross into the human population and threaten the advent of a new influenza pandemic. If a virus of a subtype represented within such a library were to cause human infections and spread between humans in the future, vaccine production could be initiated almost immediately based upon a candidate vaccine virus from the library, saving time on the path towards a pandemic vaccine. This first vaccine would not be perfectly matched to the actual outbreak virus, but might provide protection from infection and/or (severe) disease if the candidate vaccine virus and the virus of concern were antigenically related to such a degree that a protective immune response against the latter can be elicited by the former.

In contrast to highly pathogenic H5N1 viruses, for which information about their geographical distribution, evolution and their epidemiological characteristics is actively sought and usually accessible, less information is available for avian and other animal viruses of other subtypes. In addition, the number of viruses of pandemic concern is potentially large, depending on the criteria applied to the designation of viruses as relevant in pandemic preparedness activities. Therefore, viruses to be included in a library programme have to be carefully selected to ensure (i) only a manageable, limited number of candidate vaccine viruses have to be generated and (ii) these are as representative, antigenically and genetically, of animal viruses of pandemic potential as possible. The main selection criterion for inclusion in a library of strains should be antigenic representativeness, *i.e.* the chosen virus should induce antibodies that cross-react with as many viruses of a given subtype as possible. While this is hard to predict, antigenic characterisation of viruses by haemagglutination inhibition assay using post-infection ferret sera raised against a number of viruses from the same subtype usually gives an indication of where within the antigenic spectrum of a subtype any new virus is situated, and thus of how representative of other viruses it is. If done at larger scale, this analysis can be aided by antigenic cartography [40]. Unfortunately, for some subtypes of interest, very little antigenic information is available in the public domain; similarly, reagents, in particular ferret antisera, are not widely available for some subtypes. Therefore, in the absence of antigenic data, phylogenetic data describing the evolutionary relatedness of viruses may serve as a substitute in choosing a virus for inclusion in a library.

There are a number of possibilities for the type of candidate vaccine virus included in a library of strains. As most avian (and other animal) influenza A viruses, apart from some H5 and H7 viruses, are of low pathogenicity, classical reassortment with PR8 can be applied to generate a candidate vaccine virus. Reverse genetics technology can equally be used, and will normally result in viruses that are genetically and antigenically identical to those that can be generated by classical reassortment because the HA gene does not need to be modified in the case of low-pathogenic wild type viruses. In addition, wild-type viruses themselves may be used as candidate vaccine viruses in the case of low-pathogenic viruses; many viruses of pandemic concern are avian in origin, and some of these grow to high titres in embryonated hens’ eggs without further adaptation, obviating the need to manipulate the virus in order to improve growth properties [41].

We and others have started to develop such libraries of candidate vaccine viruses representing mainly avian viruses of a number of subtypes [42,43]. Table 2 shows the library of viruses available from NIBSC; viruses representing HA subtypes H5, H7, H9 and H2 are included in the library, with most candidate vaccine viruses having been derived using reverse genetics technology. These subtypes were chosen as they have all been responsible for infection and disease in humans and thus can be considered of higher pandemic risk as compared to avian viruses not known to have infected humans. The full potential of the library in relation to shortening the time between the recognition of a new virus in humans and a vaccine offering protection from this virus can only be realised if vaccine manufacturers obtain the candidate vaccine viruses during the pre-pandemic period and develop their master and working seeds ahead of a pandemic emerging; thus, vaccine production could be initiated from stored seeds whenever the need for a new vaccine is recognised or announced. Ideally, manufacturers would even produce trial lots of vaccine from (some) library viruses in order to gain experience with a new subtype. While this may appear wasteful at first glance, production of vaccine for H5N1 has shown that new subtypes can present new challenges during manufacture. Problems such as the low yield observed for some H5N1 candidate vaccine viruses might be revealed by pilot scale manufacturing. If results from this work are shared with the influenza vaccine production community as well as the laboratories generating candidate vaccine viruses, an iterative cycle of improvement of candidate vaccine viruses may be initiated, resulting in better candidate vaccine viruses and, ultimately, better pandemic preparedness for the world.

## 6. Pandemic A(H1N1)v Vaccine Development

At the end of April 2009, the system of creating vaccine viruses using reverse genetics was put to the test with the appearance of so-called swine flu in persons in Mexico and bordering states of the USA. The causative agent, an influenza H1N1 virus, was antigenically distinct from the H1N1 strains currently circulating in the human population. The WHO network of laboratories, without any need to be requested to do so, immediately began the reverse genetics process for the derivation of a 6:2 safe reassortant vaccine virus. Unfortunately, no H1N1 virus had yet been incorporated into the library of viruses of pandemic potential described above. However, the experience gleaned over the past few years on H5N1 vaccine development was of immense value and by late May 2009, candidate reverse genetics-derived vaccine viruses were available from two laboratories, CDC (USA) and NIBSC (UK) [44,45].

There were major differences in deriving a vaccine virus from pandemic H1N1v compared to H5N1 viruses. Firstly, in the case of H1N1v, the virus clearly was able to infect humans quite readily and was not adapted to growth in eggs. Secondly, the virus was not a highly pathogenic virus in the way that the current H5N1 viruses are. The HA of H1N1v does not have any extra basic amino acid residues at the HA cleavage site and so there was no requirement to delete a portion of the HA genome segment–the viral RNA could be cloned into a rescue plasmid directly with no modification. Furthermore, although the virus would need to be handled under BSL3 containment, it was nowhere as virulent as the avian H5N1 viruses, and finally, the absence of the highly pathogenic motif in the HA meant that high yielding reassortant viruses could be derived by classical reassorting as well as by reverse genetics. Consequently, in the early stages of the epidemic both reverse genetics and the classical reassorting processes were used in parallel amongst the laboratories involved, with X-179A being developed at the New York Medical College and IVR-153 at CSL, Australia [46,47]. Indeed, of the initial candidate vaccine viruses created, the most favoured was the classical reassortant X-179A. X-179A contained five genome segments from PR8 and three from the wild type A/California/07/2009 and was preferable in terms of viral protein yield to two other candidates (5.9 *vs.* 3.4–3.6 mg protein/100 eggs [unpublished observation]), including the one developed at NIBSC, NIBRG-121. All of the above was achieved during May 2009 prior to the WHO declaring level 6 pandemic influenza, which occurred on June 11, 2009.

During the development of the first vaccine viruses, following from an expert discussion, the WHO advised that vaccine production using an attenuated H1N1v vaccine virus could proceed at a BSL 2 enhanced containment level [48]. Whilst it was felt probable that a 6:2 reassortant (or in the case of X-179A, a 5:3 reassortant) between PR8 and the parental wild type virus A/California/07/2009 would be attenuated in comparison to the wild type virus, it was incumbent upon the scientists responsible to assess the virulence of H1N1v vaccine viruses empirically. Consequently, the ferret pathogenicity test as developed for H5N1 vaccine viruses was applied to the above three newly derived H1N1v candidate vaccine viruses and showed the viruses, both classical and reverse genetics derived, to be attenuated in comparison with wild type H1N1v [49]. This was still problematic for industry and it was not until the pandemic had spread sufficiently, to the extent that it was clearly circulating in the locality of vaccine manufacture plants, that the WHO lowered the level of containment to BSL 2, which is the level at which the vaccine industry manufactures seasonal flu vaccine and under which they can operate to high efficiency [50].

These first vaccine candidates were useful but produced only about one half the amount of antigen typically obtained for a seasonal H1N1 virus. In this case, unlike with the H5N1 vaccine viruses, the reason was virus growth and not HA content per virion. Thus work continued during the summer of 2009 to develop improved vaccine viruses. By August good progress had been made. Here at NIBSC, continued passage of NIBRG-121 in embryonated eggs resulted in a virus (NIBRG-121xp) that produced approximately twice as much viral protein and similar to the amount achieved with seasonal H1N1 vaccine viruses. At the same time the NYMC laboratory, by further reassortment of X-179A, produced a new reassortant (X-181) which produced viral protein to similar levels as NIBRG-121xp. These two vaccine viruses are now being used by various manufacturers to produce pandemic A(H1N1)v influenza vaccine. Table 3 lists available candidate vaccine viruses for development of pandemic H1N1v vaccines [51].

Each of the H1N1 vaccine viruses created during the summer of 2009 contained at least one mutation in the HA compared to wild type virus isolated in cell culture. These were most likely due to the need to egg-adapt the wild type H1N1v virus isolates and many of the mutations detected were similar to what has been observed in the past for the egg-adaptation of seasonal H1N1 wild type viruses [5]. Interestingly, NIBRG-121xp and X-181 had additional mutations, compared to NIBRG-121 and X-179A respectively, reflecting presumably a further adaptation of these viruses to growth in hens’ eggs. Fortunately, none of the HA mutations observed altered the antigenicity of the viruses and they remained suitable A/California/07/2009-like vaccine viruses.

To date, the H1N1v viruses have remained antigenically stable. However, these are influenza viruses and inevitably they will undergo antigenic drift and new vaccine viruses will be required to reflect these changes. In the past, the antigenic drift of human influenza A viruses has occurred in a linear fashion and as one variant gradually replaces a previous one, the strain that is most likely to dominate and cause disease has been recommended for inclusion in vaccine. In contrast, the drift of H5N1 viruses within avian species has not occurred in a linear fashion and many different antigenic variants co-exist in both the same and in disparate geographical regions. With good fortune, the H1N1v viruses will follow a linear evolution.

## Figures and Tables

**Table 1.** Available H5N1 vaccine viruses (as of September 2009) ^1^.

**Clade**	**Strain**	**Institution^2^**
**1**	A/Vietnam/1203/2004	CDC; SJ/HKU/NIAID
	A/Vietnam/1194/2004	NIBSC
	A/Cambodia/R0405050/2007	NIBSC

**2.1**	A/Indonesia/5/2005	CDC
	A/duck/Hunan/795/2002	SJ/HKU/NIAID

**2.2**	A/bar-headed goose/Qinghai/1A/2005	SJ/HKU/NIAID
	A/whooper swan/Mongolia/244/2005	SJ/NIAID
	A/chicken/India/NIV33487/2006	CDC/NIV
	A/Egypt/3300-NAMURU3/2008	CDC

**2.2.1**	A/turkey/Turkey/1/2005	NIBSC
	A/Egypt/2321/2007	CDC

**2.3.2**	A/common magpie/Hong Kong/5052/2007	SJ/HKU/NIAID

**2.3.4**	A/Anhui/1/2005	CDC
	A/Japanese white-eye/Hong Kong/1038/2006	SJ/HKU/NIAID
	A/duck/Laos/3295/2006	FDA

**4**	A/goose/Guiyang/337/2006	SJ/HKU/NIAID

**7**	A/chicken/Vietnam/NCVD-03/2008-like	CDC

1 Reproduced with permission from reference [31] (World Health Organization).

2 CDC–Centers for Disease Control and Prevention, USA; FDA–Food and Drug Administration, USA; NIAID–National Institute of Allergy and Infectious Disease, NIH, USA; NIBSC–National Institute for Biological Standards and Control, UK; NIV–National Institute of Virology, India; SJ–St Jude Children’s Research Hospital, USA; HKU–University of Hong Kong, China Hong Kong SAR.

**Table 2.** NIBSC library of pandemic vaccine viruses.

**Subtype**	**Wild-type virus**	**Candidate vaccine virus**
**H5N3**	A/duck/Singapore-Q/F119-3/1997	ARIV-1^1^
**H5N1 (clade 1)**	A/Hong Kong/213/2003	NIBRG-12^2^
**H5N1 (clade 1)**	A/Vietnam/1194/2004	NIBRG-14
**H5N1 (clade 1)**	A/Cambodia/R0405050/2007	NIBRG-88
**H5N1 (clade 2.2.1)**	A/turkey/Turkey/1/2005	NIBRG-23
**H7N1**	A/turkey/Italy/3889/1999 (low path)	wt
**H7N3**	A/mallard/Netherlands/12/2000	NIBRG-60
**H7N1**	A/mallard/Netherlands/12/2000	NIBRG-63
**H7N2**	A/New York/107/2003	NIBRG-109
**H9N2**	A/Hong Kong/1073/1999 (G1 lineage)	wt
**H9N2**	A/chicken/Hong Kong/G9/1997(G9 lineage)	NIBRG-91
**H2N3**	A/mallard/England/727/2006	NIBRG-107

1 A classically derived reassortant.

2 All vaccine viruses coded ‘NIBRG’ are reverse genetics derived.

**Table 3.** Available pandemic H1N1v 2009 vaccine viruses (as of October 2009) ^1^.

Parent virus	Vaccine virus	Developing institute ^2^	Available since
A/California/07/2009	Wild type	CDC	May 09
	X-179A ^cl^	NYMC	27 May 09
	IVR-153 ^cl^	CSL	04 June 09
	X-181 ^cl^	NYMC	14 Sept 09
	X-181A ^cl^	NYMC	14 Sept 09
	NIBRG-121 ^rg^	NIBSC	27 May 09
	NIBRG-121xp ^rg^	NIBSC	06 Aug 09
A/California/04/2009	Wild type	CDC	May 09
	CBER-RG2 ^rg^	CBER	19 June 09
A/Texas/5/2009	Wild type	CDC	May 09
	IDCDC-RG15 ^rg^	CDC	27 May 09
	IDCDC-RG20 ^rg^	CDC	22 July 09
A/England/195/2009	Wild type	NIBSC	May 09
	NIBRG-122 ^rg^	NIBSC	22 July 09
A/Texas/5/2009 & A/New York/18/2009	IDCDC-RG18 ^rg^	CDC	22 July 09
A/New York/18/2009	IDCDC-RG22 ^rg^	CDC	22 July 09

1 Reproduced with permission from reference [51] (World Health Organization).

2 CBER–Centers for Biologics Evaluation and Research, USA; CDC–Centers for Disease Control and Prevention, USA; CSL–Commonwealth Serum Laboratories, Australia; NYMC–New York Medical College, USA; NIBSC–National Institute for Biological Standards and Control, UK.

cl classical reassortant

rg reverse genetics derived

## References

[1] Wood JM, Williams MS, Nicholson KG, Webster RG, Hay AJ (1998). History of inactivated vaccines. Textbook of Influenza.

[2] Furminger IGS, Nicholson KG, Webster RG, Hay AJ (1998). Vaccine production. Textbook of Influenza.

[3] Burnet FM, Bull DR (1943). Changes in influenza virus associated with adaptation to passage in chick embryos. Aust J Exp Biol Med Sci.

[4] Schild GC, Oxford JS, De Jong JC, Webster RG (1983). Evidence for host-cell selection of influenza virus antigenic variants. Nature (London).

[5] Robertson JS (1993). Clinical influenza virus and the embryonated hen's egg. Reviews in Medical Virology.

[6] Kilbourne ED, Murphy JS (1960). Genetic studies of influenza viruses: I. Viral morphology and growth capacity as exchangeable genetic traits. Rapid *in ovo* adaptation of early passage Asian strain isolates by combination with PR8. J Exp Med.

[7] Kilbourne ED (1969). Future influenza vaccines and the use of genetic recombinants. Bull WHO.

[8] Kilbourne ED, Schulman JL, Schild GC, Schloer G, Swanson J, Bucher D (1971). Correlated studies of a recombinant influenza virus vaccine. I. Derivation and characterization of the virus and vaccine. J Infect Dis.

[9] Burnet FM, Lind PE (1951). A genetic approach to variation in influenza viruses. 3. Recombination of characters in influenza virus strains used in mixed infections. J gen Microbiol.

[10] McGeoch D, Fellner P, Newton C (1976). Influenza virus genome consists of eight distinct RNA species. Proc Natl Acad Sci USA.

[11] Baez M, Palese P, Kilbourne ED (1980). Gene composition of high-yielding influenza vaccine strains obtained by recombination. J Infect Dis.

[12] Enami M, Luytjes W, Krystal M, Palese P (1990). Introduction of site-specific mutations into the genome of influenza virus. Proc Natl Acad Sci USA.

[13] Fodor E, Devenish L, Engelhardt OG, Palese P, Brownlee GG, García-Sastre A (1999). Rescue of influenza A virus from recombinant DNA. J Virol.

[14] Neumann G, Watanabe T, Ito H, Watanabe S, Goto H, Gao P, Hughes M, Perez DR, Donis R, Hoffmann E, Hobom G, Kawaoka Y (1999). Generation of influenza A viruses entirely from cloned cDNAs. Proc Natl Acad Sci USA.

[15] Nicolson C, Major D, Wood JM, Robertson JS (2005). Generation of influenza vaccine viruses on Vero cells by reverse genetics: an H5N1 candidate vaccine strain produced under a quality system. Vaccine.

[16] Hoffmann E, Neumann G, Kawaoka Y, Hobom G, Webster RG (2000). A DNA transfection system for generation of influenza A virus from eight plasmids. Proc Natl Acad Sci USA.

[17] Neumann G, Fujii K, Kino Y, Kawaoka Y (2005). An improved reverse genetics system for influenza A virus generation and its implications for vaccine production. Proc Natl Acad Sci USA.

[18] Ozawa M, Goto H, Horimoto T, Kawaoka Y (2007). An adenovirus vector-mediated reverse genetics system for influenza A virus generation. J Virol.

[19] Klenk HD, Rott R, Orlich M, Blödorn J (1975). Activation of influenza A viruses by trypsin treatment. Virology.

[20] Wood GW, McCauley JW, Bashiruddin JB, Alexander DJ (1993). Deduced amino acid sequences at the haemagglutinin cleavage site of avian influenza A viruses of H5 and H7 subtypes. Arch Virol.

[21] Steinhauer DA (1999). Role of hemagglutinin cleavage for the pathogenicity of influenza virus. Virology.

[22] Subbarao K, Chen H, Swayne D, Mingay L, Fodor E, Brownlee G, Xu X, Lu X, Katz J, Cox N, Matsuoka Y (2002). Evaluation of a genetically modified reassortant H5N1 influenza A virus vaccine candidate generated by plasmid-based reverse genetics. Virology.

[23] World Health Organization (WHO) (2005). Availability of H5N1 Prototype Strains for Influenza Pandemic Vaccine Development.

[24] Office Internationale des Epizooties (OIE) (2009). Avian Influenza. Manual of diagnostic tests and vaccines for terrestrial animals.

[25] World Health Organisation (WHO) Production of Pilot Lots of Inactivated Influenza Vaccines from Reassortants Derived from Avian Influenza Viruses. Interim Biosafety Risk Assessment. Annex 1, Testing for Attenuation of Influenza Vaccine Strains in Mammals.

[26] Bresson J, Perronne C, Launay O, Gerdil C, Saville M, Wood JM, Höschler K, Zambon MC (2006). Safety and immunogenicity of an inactivated split-virion influenza A/Vietnam/1194/2004 (H5N1) vaccine: phase I randomised trial. Lancet.

[27] Leroux-Roels I, Borkowski A, Vanwolleghem T, Dramé M, Clement F, Hons E, Devaster J, Leroux-Roels G (2007). Antigen sparing and cross-reactive immunity with an adjuvanted rH5N1 prototype pandemic influenza vaccine: a randomised controlled trial. Lancet.

[28] Qiu YZ, Yin WD Safety and immunogenicity of Sinovac's prototype pandemic influenza H5N1 vaccines: a review on clinical trials. Influenza Other Respi Viruses.

[29] European Medicines Agency CHMP Guideline on Dossier Structure and Content for Pandemic Influenza Vaccine Marketing Authorisation Application (revision).

[30] European Medicines Agency CPMP Guideline on Submission of Marketing Authorisation Applications for Pandemic Influenza Vaccines through the Centralised Procedure.

[31] World Health Organisation (WHO) Antigenic and Genetic Characteristics of Influenza A(H5N1) Viruses and Candidate Vaccine Viruses Developed for Potential Use in Human Vaccines.

[32] Wood JM, Nicholson KG, Webster RG, Hay AJ (1998). Standardisation of inactivated influenza vaccines. Textbook of Influenza.

[33] NIBSC, HPA Current Availability from NIBSC of Candidate Influenza Vaccine Viruses (H5N1) and SRD Reagents for Vaccine Standardisation.

[34] Harvey R, Wheeler JX, Wallis CL, Robertson JS, Engelhardt OG (2008). Quantitation of haemagglutinin in H5N1 influenza viruses reveals low haemagglutinin content of vaccine virus NIBRG-14 (H5N1). Vaccine.

[35] Horimoto T, Murakami S, Muramoto Y, Yamada S, Fujii K, Kiso M, Iwatsuki-Horimoto K, Kino Y, Kawaoka Y (2007). Enhanced growth of seed viruses for H5N1 influenza vaccines. Virology.

[36] Adamo JE, Liu T, Schmeisser F, Ye Z (2009). Optimizing viral protein yield of influenza virus strain A/Vietnam/1203/2004 by modification of the neuraminidase gene. J Virol.

[37] Subbarao K, Joseph T (2007). Scientific barriers to developing vaccines against avian influenza viruses. Nat Rev Immunol.

[38] Van Reeth K (2007). Avian and swine influenza viruses: our current understanding of the zoonotic risk. Vet Res.

[39] de Wit E, Fouchier RAM (2008). Emerging influenza. J Clin Virol.

[40] Smith DJ, Lapedes AS, de Jong JC, Bestebroer TM, Rimmelzwaan GF, Osterhaus AD, Fouchier RA (2004). Mapping the antigenic and genetic evolution of influenza virus. Science.

[41] Nicholson KG, Colegate AE, Podda A, Stephenson I, Wood J, Ypma E, Zambon MC (2001). Safety and antigenicity of non-adjuvanted and MF59-adjuvanted influenza A/Duck/Singapore/97 (H5N3) vaccine: a randomised trial of two potential vaccines against H5N1 influenza. Lancet.

[42] Kida H, Sakoda Y (2006). Library of influenza virus strains for vaccine and diagnostic use against highly pathogenic avian influenza and human pandemics. Dev Biol Basel.

[43] Karron RA, Callahan K, Luke C, Thumar B, McAuliffe J, Schappell E, Joseph T, Coelingh K, Jin H, Kemble G, Murphy BR, Subbarao K (2009). A live attenuated H9N2 influenza vaccine is well tolerated and immunogenic in healthy adults. J Infect Dis.

[44] World Health Organisation (WHO) Availability of a Candidate Reassortant Vaccine Virus for the Novel Influenza A (H1N1) Vaccine Development.

[45] World Health Organisation (WHO) Availability of a Candidate Reassortant Vaccine Virus for the Novel Influenza A (H1N1) Vaccine Development.

[46] World Health Organisation (WHO) Availability of a Candidate Reassortant Vaccine Virus for the Novel Influenza A (H1N1) Vaccine Development.

[47] World Health Organisation (WHO) Availability of a Candidate Reassortant Vaccine Virus for the Novel Influenza A (H1N1) Vaccine Development.

[48] World Health Organisation (WHO) Update of WHO Biosafety Risk Assessment and Guidelines for the Production and Quality Control of Human Influenza Pandemic Vaccines.

[49] World Health Organisation (WHO) Biocontainment Requirements for Vaccine Production from and Quality Control of the Reassortant Vaccine Candidate Viruses IDCDC-RG15, NIBRG-121 and X-179A.

[50] World Health Organisation (WHO) WHO Biosafety Risk Assessment and Guidelines for the Production and Quality Control of Human Influenza Pandemic Vaccines: Update 23 July 2009.

[51] World Health Organisation (WHO) Summary of Available Candidate Vaccine Viruses for Development of Pandemic (H1N1) 2009 Virus Vaccines.

